# EFFECT OF COMMUNITY HEALTHCHOICES ON PARTICIPANT QUALITY OF LIFE AND PSYCHOLOGICAL WELL-BEING

**DOI:** 10.1093/geroni/igac059.562

**Published:** 2022-12-20

**Authors:** Howard Degenholtz

**Affiliations:** University of Pittsburgh, Pittsburgh, Pennsylvania, United States

## Abstract

A stratified random sample of participants was interviewed in each region of Pennsylvania during each phase of the implementation. In addition, comparison groups were interviewed from the third implementation region. This allowed us to draw causal inferences regarding the effect of the program on participant quality of life and psychological well-being. The sample was stratified to represent: people age 21-59 and those over age 60 who receive home and community based services (HCBS), plus people age 21 and older who are dually eligible for Medicaid and Medicare but do not use long-term services and supports. We found that engagement in preferred activities both inside and outside the home increased among people who used HCBS and those who did not. In addition, psychological well-being improved slightly while the prevalence of depressive symptoms declined. In general, the implementation of Community HealthChoices appears to have led to improvements in several measures of well-being.

